# Impact of classroom-based MASK-ED™ (KRS simulation) on physiotherapy student clinical performance: a randomized cluster trial

**DOI:** 10.1186/s12909-022-03467-8

**Published:** 2022-06-02

**Authors:** Tayne Ryall, Elisabeth Preston, Niruthikha Mahendran, Bernie Bissett

**Affiliations:** 1 Faculty of Health, University of Canberra, ACT, Canberra, Australia; 2 Canberra Hospital, Canberra Health Services, ACT, Canberra, Australia; 3 School of Health and Rehabilitation Sciences, University of Queensland, Brisbane, QLD Australia

**Keywords:** Simulation training, Education, Students, Universities, Physical therapy, Randomized control trial

## Abstract

**Background:**

In physiotherapy there is a growing body of literature exploring the benefits simulation could have in the university-setting, prior to the commencement of work-integrated learning. MASK-ED™ simulation is one form of simulation that could be beneficial for student learning and improve performance in the clinical setting. MASK-ED™ simulation involves an educator donning a silicone mask and portraying a patient role that has been specifically developed to meet learning objectives.

**Objective:**

To evaluate the effectiveness of MASK-ED™ simulation compared to role-play with peers for training pre-clinical physiotherapy students.

**Methods:**

A single-centre, single-blind, cluster randomized trial with concealed allocation, between group post-measures, and intention-to-treat analysis was conducted at an Australian university between February 2018 – January 2021. Participants were 144 physiotherapy students, cluster randomized by tutorial groups (exp *n* = 70, con *n* = 74), undertaking their neurological curricula. The experimental group was exposed to MASK-ED™ simulation in five out of a potential thirty-two tutorials (16%) whilst the control continued with role-play with peers. The primary outcome measure was Assessment of Physiotherapy Practice scores from the students’ rehabilitation work-integrated learning clinical placement. These were compared between the experimental and control groups using Mann–Whitney U tests. Secondary outcome measures include practical and written examination scores. These were compared between groups via independent t-tests. Participant satisfaction surveys were also administered to the experimental group.

**Results:**

One hundred thirty-two participants’ (exp *n* = 62, con *n* = 72) results were analyzed. There were no significant differences between the experimental and control groups for Assessment of Physiotherapy Practice scores (*p* = 0.699–0.995). There were no significant differences found between the groups, across the secondary outcome measures. Participants found MASK-ED™ simulation was somewhat helpful for preparing them for clinical practice, however felt that a group setting was not as effective as a one-on-one encounter would have been.

**Conclusions:**

MASK-ED™ simulation was no more effective than role-play with peers in preparing physiotherapy students for work-integrated learning. The influence of the design of simulation on effective learning and the number of classroom-based simulation encounters required to impact clinical performance requires further investigation.

**Supplementary Information:**

The online version contains supplementary material available at 10.1186/s12909-022-03467-8.

## Background

Physiotherapy students are required to learn a great variety of complex skills. These include both technical and non-technical skills, both of which are taught more effectively in an authentic context [1]. Work integrated learning allows physiotherapy students to apply these skills in a highly variable and complex environment and allows clinical educators to monitor and assess students’ competence over a sufficient period of time and across a range of patient types [2]. However, in some instances, students are not adequately prepared for work integrated learning, and this can lead to anxiety and distress for students, concerns regarding patient safety, and can be stressful and burdensome for clinical educators [3–5].

Classroom-based simulation may be an effective strategy for improving student performance during clinical practice. Health professional students commonly report finding various forms of simulation to be enjoyable and effective in building their confidence and/or in preparing them for the clinical environment [6–15]. The simulation types include high-fidelity patient simulators, standardised patients, part-task trainers, virtual reality, and computer-based simulations (e.g., virtual patients and serious games). Simulation in physiotherapy student education has predominantly focused on simulation-based assessments, or replacing work integrated learning [16–20]. Research into classroom-based simulation in physiotherapy has demonstrated that students both enjoy classroom-based simulation, such as standardised patients and peer simulation, and perceive it better prepares them from work integrated learning than peer-role play [14, 15, 21]. Two systematic reviews have found that simulation-based learning, either replacing clinical time or as classroom-based learning, is mostly well received by physiotherapy students, however further rigorous research is required to determine how simulation should be implemented into university curriculum and the effect it can have on clinical performance [22, 23].

MASK-ED™ simulation has been used as part of physiotherapy classroom-based education since 2013 [24]. MASK-ED™ simulation commenced in nursing education in 2010 at Central Queensland University, Australia and involves an educator donning a silicone mask and taking on the role of a patient with a predetermined history, specific to the learning objectives [25]. Health Professional students enjoy classroom-based MASK-ED™ simulation and perceive that it helps to prepare them for work integrated learning [26, 27]. Physiotherapy students specifically have reported that MASK-ED™ simulation helps them to feel better prepared for work integrated learning than practising with peers, particularly regarding professionalism, and developing skills such as communication, rapport, and empathy [24]. The impact that MASK-ED™ simulation has on clinical performance has not been examined. Therefore, the research questions for this study were:

1. Is MASK-ED™ simulation more effective than role-play with peers in improving physiotherapy student clinical performance during work integrated learning?
2. Which components, if any, of physiotherapy student clinical performance does MASK-ED™ simulation affect?
3. What are students’ perceptions of MASK-ED™ simulation?

## Methods

### Design

The study was a single-centre, single-blind, cluster randomized trial with concealed allocation, between group post-measures, and intention-to-treat analysis. The aim was to compare the use of MASK-ED™ simulation with role play with peers, to determine the effect on physiotherapy students’ clinical performance during their work integrated learning placements. In designing the study the investigating team took into consideration the best practice design features of simulation-based education outlined by McGaghie et al. of feedback on performance, deliberate practice, curriculum integration, outcome measurement, simulation fidelity, skills acquisition and maintenance, mastery learning, transfer to practice, team training, high-stakes testing, instructor training, educational and professional context [28]. The primary outcome was clinical performance, measured using the Assessment of Physiotherapy Practice (APP), completed at the end of the clinical placement based on the students’ performance during work integrated learning. The full study protocol has been published previously [29].

### Study setting and participants

The study took place at an Australian university as part of the neurological units of study undertaken by physiotherapy students enrolled in either a bachelor or a graduate entry master program (2018–21). The students were in either their third year of a four-year bachelor’s degree or their first year of a two-year graduate-entry master’s degree. Students commence their work integrated learning between 2 weeks to 6 months after successfully completing their neurological curriculum over two semesters. Tutorial groups were cluster randomized to receive either the experimental (MASK-ED™) or the control intervention (role play). Baseline characteristics were collected, including grade point averages, scored between 0 and 7, where < 4 is equivalent to a fail and 7 is equivalent to a high distinction. Tutorials were conducted in a purpose-built physical skills lab designed to replicate a rehabilitation gymnasium environment.

### Intervention

**Fig. 1:**
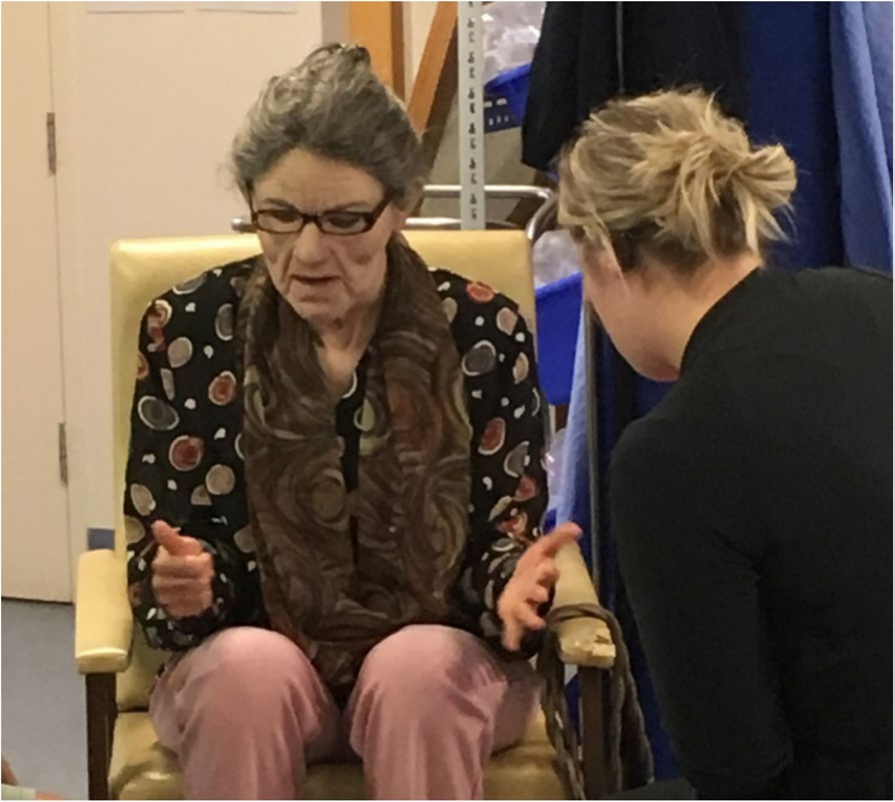
MASK-ED™ character “Joyce Fullerton” with a student during a tutorial

The experimental group participated in MASK-ED™ simulation during five out of a potential 32 tutorials (16%) as well as usual teaching. The MASK-ED™ character “Joyce” (Fig. 1), who is portrayed by the same educator every session, was introduced to all of the students in a previous unit of study, with a specific past medical and social history [24]. A second educator was present during the experimental tutorials to assist the masked educator and the students. The four experimental groups were taught by one tutor, and the four control groups by another tutor [29].

Prior to the experimental group commencing their tutorials with the MASK-ED™ character they were shown a short video of her being interviewed by one of the lecturers outlining that she had suffered a stroke several weeks prior and her ongoing rehabilitation goals. In small groups, of 3–5 students, students were given opportunities to ask the MASK-ED™ character questions and practise assessment and intervention skills that were covered in lecture content, such as assessment and interventions targeting upper limb, balance, falls prevention, and gait retraining. The specific learning objectives for each of the five sessions have been outlined in Table 1. Whilst each of the intervention groups were required to interact with the MASK-ED™ character and demonstrate assessment and interventions skills, it was not compulsory for each student to interact with the MASK-ED™ character. This was a pragmatic decision to meet the resource and time constraints of academic teaching and learning, thereby ensuring a generalisable intervention. Also, MASK-ED™ simulation recommends an educator should don the mask for a maximum of 15 minutes and this was considered in the tutorial design.

**Table 1:** Tutorial learning outcomes

Tutorial 1: Reaching and manipulation • Identify common adaptive strategies made by people after stroke make when undertaking reaching and manipulation tasks • Assess common reaching and manipulation tasks • Demonstrate training strategies for reaching and manipulation when patients can reach for and manipulate objects but with difficulty • Demonstrate promoting flexibility of performance in reaching and manipulation when patients can reach for and manipulate objects but with difficulty Tutorial 2: Walking • Understand how particular impairments contribute to activity limitations in walking after stroke • Demonstrate assessment and measurement of walking • Demonstrate training walking when patients can walk but with difficulty • Demonstrate promoting flexibility of performance in walking when patients can walk but with difficulty Tutorial 3: Upper limb assessment and intervention • Demonstrate a comprehensive upper limb assessment of impairments and activity– reaching for a cup (with MASK-ED • Develop and demonstrate an intervention for reaching for a cup based on upper limb assessment findings Tutorial 4: Managing falls • Describe the purpose and use of a falls diary • Describe methods of compensating for poor balance to prevent falls • Demonstrate training strategies for “getting up from the floor” Tutorial 5: Clinical reasoning in the physiotherapy management of cerebellar ataxia • Develop and demonstrate strategies for flexibility of performance in someone with cerebellar ataxia for: a) Walking with a dual task b) Walking over obstacles c) Walking with change of direction/speed d) Hanging washing on a line e) Shuffling cards f) Manipulation of nuts and bolts	

The tutorial structure for the intervention sessions followed one of two formats:

- briefing, followed by four to six stations including a station for planning/debriefing, and a station for interaction with the MASK-ED™ character. The other two to four stations involving activities relevant to learning outcomes; and/or
- briefing, planning, and then small group interactions with the MASK-ED™ character in front of the class followed by a class debriefing.

The tutorials for the control group followed the same format as the experimental group, with the control participants performing the same activities in small groups but practising on one another rather than with the MASK-ED™ character. Through practising assessment and intervention skills the students were also able to practise their manual handling skills, communication, and professionalism.

The control group received usual teaching only, including role-play with peers. For the purpose of this study, we defined role play with peers as practicing skills with each other, with no script or guidance being provided to the students about portraying the role of a patient, and no elements of simulation used. Usual teaching involved weekly one-hour lectures in a large group (approximately 90 students) and two 2.5-hour tutorials in small groups (≤24 students) over 10 weeks. The tutorials were a combination of video-taped practical skill demonstrations and student practice with peers, and case-based learning [29].

### Outcome measures

The primary outcome was clinical performance measured using the Assessment of Physiotherapy Practice (APP) [30]. The APP is the standardised assessment tool used across Australia to assess physiotherapy students on their work integrated learning placements, that is both reliable and valid [2, 31]. The overall APP results (out of 80) and the sub-components *Communication* (Criterion 5: verbal and non-verbal, minimum of 0 to maximum of 4), *Professionalism* (minimum of 0 to maximum 16), *Assessment* (minimum of 0 to maximum 12), and *Intervention* (minimum of 0 to maximum of 20) from the students’ five-week rehabilitation work integrated learning placement were collected [29]. These subcomponents were selected due to the learning objectives of the tutorials where the MASK-ED™ character was introduced and previous self-reported beliefs that MASK-ED™ simulation helps to develop communication skills and professionalism [24].

Other secondary outcomes were students’ performance of clinical skills measured during practical examinations (out of 25), and their written examination marks at the end of each their units of study, prior to their rehabilitation work-integrated learning placement [29]. The written and practical examinations are entirely case based and assess learning outcomes related to assessment, measurement, intervention, interpretation and knowledge, aimed at determining the students’ readiness for work integrated learning.

Students in the experimental group were also asked to complete a survey at the end of the five intervention tutorials to examine their perception of MASK-ED™ simulation. The questions consisted of 14 questions with responses on a 5-point Likert scale where 0 = Very unhelpful and 4 = Very helpful, and four open-ended questions (Table 2). The survey has been used in previous research about MASK-ED™ [24]. Face and content validity were completed through discussion amongst the research teams.

**Table 2:** Survey questions for experimental participants

	Very unhelpful (0)	Somewhat unhelpful (1)	Neutral (2)	Somewhat helpful (3)	Very helpful (4)
Confidence engaging with an older person					
Developing rapport and empathy with patients					
Manual handling skills					
Communicating with an older patient					
Explaining treatments without using jargon					
Ability to step into the physiotherapist role					
Ability to apply theory to practice					
Interest / engagement with the material covered in the unit					
Remembering practical lessons from the classroom					
Self-reflection and learning from mistakes in a safe environment					
Potential to learn from other students’ experiences (peer learning)					
Ability to give / receive feedback					
Readiness to undertake the practical exam					
Readiness to undertake clinical placement					
Do you think that MASK-ED™ simulation has been beneficial in any other way to enhance learning? Please describe.					
In the classroom, is MASK-ED™ simulation more valuable for students than just practicing on each other? Please explain why / why not:					
Are there any negative aspects to including MASK-ED™ simulation in the Physiotherapy classroom?					
On balance, do you think we should continue MASK-ED™ simulation as a feature of the UC Physiotherapy curriculum? Please explain why / why not:					

### Data analysis

Based on sample size calculations, 120 students were required to detect a 0.5/4 mark difference (12.5% change on the primary outcome) with 80% power at a two-tailed significance level of 0.05 [29]. APP *Communication*, *Professionalism*, *Assessment*, *Intervention* and Overall results were compared between groups using Mann–Whitney U tests. Practical and written examination marks were compared as mean differences (95% CI) using independent t-tests. Intention-to-treat analysis was undertaken. All analysis was calculated in SPSS (Version 27). Concurrent triangulation design was used for collection and analysis of the survey results [32]. The quantitative and qualitative data was collected at the same time and analysed concurrently. Likert scores from participant surveys were quantitatively analysed, and the open-ended questions were themed using a Grounded Theory approach, whereby themes were inductively deduced as the data was being analysed and comparisons were made looking for similarities and differences across the students’ comments [33]. Themes were developed by one researcher and then cross checked by a second researcher.

## Results

### Flow of participants through the study

**Fig. 2:**
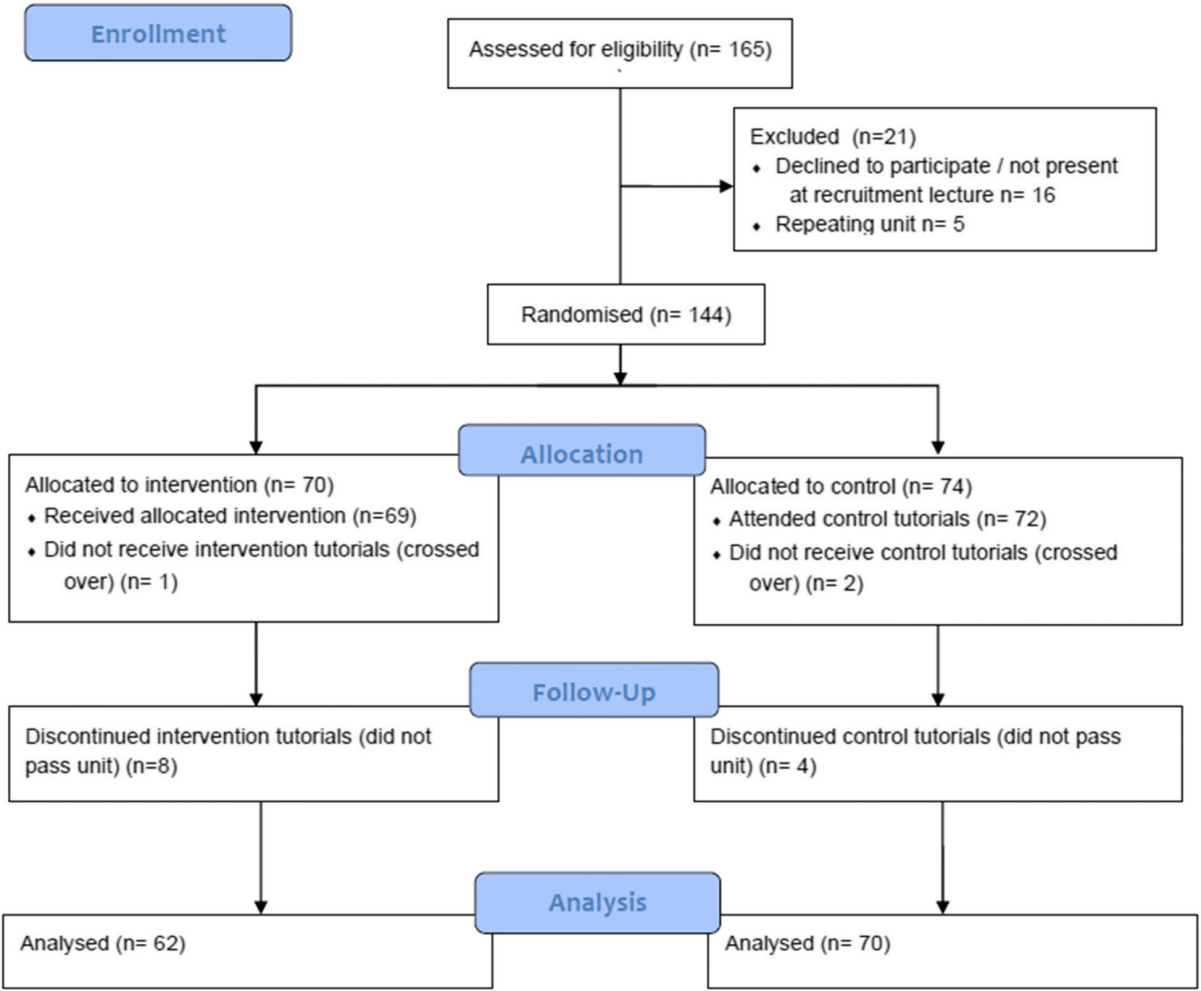
Participant flow through study

Recruitment of the first cohort of students took place in Semester 1 February 2018 with the final work integrated learning placement completed in January 2021. A total of 144 students, enrolled in 8 tutorial groups, were randomized to the experimental (*n* = 70) and control (*n* = 74) groups. Participant flow through the study is outlined in Fig. 2, and participant characteristics are presented in Table 3. Twelve students failed to meet the requirements to move through the curriculum in the outlined timeframe and therefore were considered dropouts. All students who dropped out of the study had GPAs of < 5.4, eight were from the experimental group. The principles of analysis were applied as per the CONSORT Statement 2010 [34] for randomized trials and therefore the two students that crossed over from the control group into the experimental group between units were treated as control participants. One student was randomized into the experimental group, but attended all control tutorials, they were treated as an experimental participant for data analysis.

**Table 3:** Baseline characteristics of participants

Characteristic	Randomized (*n* = 132)	
	Exp (*n* = 62)	Con (*n* = 70)
Participants		
Age *(yr)*, mean (SD)	27 (5)	28 (5)
Gender, n females (%)	32 (52%)	43 (61%)
Degree category, n (%)		
Undergraduate	49 (79%)	52 (74%)
Postgraduate	13 (21%)	18 (26%)
GPA, mean (SD) (0–7)	5.69 (0.67)	5.73 (0.57)
Attendance rate	95%	91%

*Exp* experimental group, *Con* control group, *GPA* Grade Point Average

### Effect of MASK-ED™ simulation on clinical performance during work integrated learning

The introduction of MASK-ED™ simulation into five tutorials across two semesters of a neurological physiotherapy curriculum had no significant effect on student performance during work integrated learning, when compared to usual teaching (Table 4). Six students from the control group (9%) and three students from the experimental group (5%) failed to demonstrate competency on the APP and were required to repeat their rehabilitation work integrated learning placement.

**Table 4:** Mean (SD) and mean difference (95% CI) and significance for Assessment of Physiotherapy Practice

Outcome measure	Post Intervention			
	Exp (*n* = 62) Mean (SD)	Con (*n* = 70) Mean (SD)	Difference between groups Mean (95% CI)	Significance^a^
APP Communication (verbal and non-verbal) Out of 4	2.87 (0.713)	2.89 (0.877)	0.015 (−0.26 to 0.29)	0.799
APP Professionalism Out of 16	12.24 (2.546)	12.13 (2.874)	−0.113 (−1.05 to 0.83)	0.985
APP Assessment Out of 12	7.90 (1.657)	7.97 (1.857)	0.068 (−0.54 to 0.68)	0.699
APP Intervention Out of 20	13.04 (3.130)	13.06 (3.396)	0.013 (−1.12 to 1.14)	0.730
APP Total Out of 80	54.71 (11.416)	54.24 (12.738)	−0.463 (−4.65 to 3.72)	0.995

*Exp* experimental group, *Con* control group, *APP* Assessment of Physiotherapy Practice, *SD* Standard deviation, *CI* confidence interval

^a^Significance determined by Mann-Whitney U test

### Effect of MASK-ED™ simulation on specific aspects of student performance

There was no significant difference between the experimental and control groups for *Communication* (*p* = 0.799), *Professionalism* (*p* = 0.985), *Assessment* (*p* = 0.699), *Intervention* (*p* = 0.730), or *Total APP* (*p* = 0.995) scores during clinical placement (Table 4). Across the practical and written examinations for semester 1 and semester 2 there were no significant differences in performance between the experimental and control groups (Table 5).

**Table 5:** Mean (SD) and mean difference (95% CI) and significance for practical and written examinations

Outcome measure	Exp (*n* = 62) Mean (SD)	Con (*n* = 70) Mean (SD)	Difference between groups Mean (95% CI)	Significance
Practical Examination 1 (Semester 1) Out of 25	17.79 (4.60)	17.29 (4.44)	0.03 (−2.06 to 1.06)	0.529
Practical Examination 2 (Semester 1) Out of 25	20.25 (2.88)	19.83 (2.97)	−0.50 (−1.43 to 0.59)	0.411
Practical Examination 1 (Semester 2) Out of 15	11.81 (2.01)	11.87 (2.14)	0.05 (−0.66 to 0.77)	0.882
Practical Examination 2 (Semester 2) Out of 20	15.51 (1.67)	15.20 (2.03)	−0.31 (− 0.95 to 0.33)	0.343
Written Examination (Semester 1) Out of 50	32.70 (4.46)	33.21 (4.78)	0.51 (−1.09 to 2.10)	0.533
Written Examination (Semester 2) Out of 40	28.48 (3.51)	27.94 (4.57)	−0.55 (−1.96 to 0.33)	0.445

*Exp* experimental group, *Con* control group, *SD* Standard deviation, *CI* confidence interval

### Student perception of MASK-ED™ simulation

The response rate was 52% for the survey by participants in the experimental group. MASK-ED™ simulation was deemed to be *somewhat helpful* in all areas surveyed (Table 6). The majority of respondents (82%) felt that MASK-ED™ simulation should be continued, however with suggestions for improvement. For the 18% who felt that it should not be continued, their reasons for discontinuing were similar to the suggested areas of improvements.

**Table 6:** Survey responses for experimental participants

Question	Results Mean (SD)
Confidence engaging with an older person	3 (0.5)
Developing rapport and empathy with patients	3 (0.4)
Manual handling skills	3 (0.9)
Communicating with an older patient	3 (0.5)
Explaining treatments without using jargon	3 (0.5)
Ability to step into the physiotherapist role	3 (0.4)
Ability to apply theory to practice	3 (0.8)
Interest / engagement with the material covered in the unit	3 (0.8)
Remembering practical lessons from the classroom	3 (0.9)
Self-reflection and learning from mistakes in a safe environment	3 (0.5)
Potential to learn from other students’ experiences (peer learning)	3 (1.2)
Ability to give / receive feedback	3 (0.5)
Readiness to undertake the practical exam	3 (0.8)
Readiness to undertake clinical placement	3 (1.1)

Very unhelpful = 0; Somewhat unhelpful = 1; Neutral = 2; Somewhat helpful = 3; Very helpful = 4

The themes identified from the open-ended questions were:

- *Improved learning environment:* students felt working with the MASK-ED™ character was an enjoyable way to learn that helped them to consolidate their theory and practical skills. It allowed the students to learn from their mistakes and ensured they were accountable for their preparation for class and learning during class. They felt if they were underprepared for the tutorials then they were not ready to participate in MASK-ED™ simulation. The following quotes demonstrate these points:

- *“it is an engaging fun way to learn”*
- *“it allows us to practice our practical skills, critical thinking and manual handling at a greater level when compared to practicing on each other”*

b) *Realism:* some students felt that the MASK-ED™ character was more realistic than practising on each other, and that it helped them to feel like a physiotherapist. While it was acknowledged that working with the MASK-ED™ character meant activities took longer, they felt this was worthwhile and more realistic to clinical practice. The theme of realism was demonstrated through the following quotes:

- *“it’s like going on placement, but not!”*
- *“it allows us to act as a physiotherapist to a ‘real’ patient”*
- “*it takes a bit longer to get through the activities, but this is preparation for clinical placement and working with patients*”

Conversely, some students reported that they MASK-ED™ character learnt too quickly and was therefore unrealistic.

iii) *The group environment hindered time to practise with the MASK-ED™ character:* most notably most students felt they did not have enough time practising with the MASK-ED™ character and felt and that the group environment did not allow for as much individual practise time. This point is highlighted by the following quotes:

- “*time for preparation hinders time for learning*”
- “*MASK-ED was more valuable when done with one-on-one experiences rather than group or class interactions*”

iv) *Timing and incorporation into the curriculum:* students felt that further consideration was required about the ideal timing of the character and the link to the curriculum.

The themes identified are outlined further in Table 7, beneficial components, and Table 8, areas for improvement.

**Table 7:** Components of MASK-ED™ simulation that were found to be beneficial

*Improved learning environment:* • Consolidation of what was learnt • Helped to visualise how to set-up interventions, equipment and space required, and how to “think outside the box” • Helped during the practical examinations as students were able to visualise Joyce instead of the student in front of them • Helped to improve safety with patients and decrease use of jargon • Learning from mistakes • Encouraged accountability, more so than when practising with each other • Helped to motivate to practise within the tutorial times • Good way to add a different teaching style • Allows for practical learning rather than rote learning *Realism:* • MASK- ED™ character replicated symptoms of actual patients • More realistic / real life practice situations • Took longer, but this felt more realistic in terms of working with older patients in a clinical environment

**Table 8:** Suggested areas of improvement for MASK-ED™ simulation

*Group environment hindered time to practise* • Student to MASK-ED™ character ratio meant not enough time to practise • Better when one-on-one • Too much down time when done in groups • More confident students would take control • Limit group numbers to four • Split up groups of friends • Felt that when not interacting with the MASK-ED™ character were not learning • Not everyone was able to practise with the MASK-ED™ character *Timing of the MASK-ED™ character* • Less helpful for new skill acquisition • More valuable at the beginning of the curriculum • Need to relate back to the evidence *Realism* • Frustrating as it can feel exaggerated • MASK-ED™ character learnt too quickly and therefore wasn’t realistic • Different MASK-ED™ characters for the different tutorials	

## Discussion

Based on this randomized cluster trial, exposure to a MASK-ED™ character, designed to meet the learning outcomes of a neurological physiotherapy curriculum, on five occasions did not improve work integrated learning results compared with usual role play. There were no differences between the experimental and control groups in academic results. The survey findings from the experimental group did show that the students enjoyed MASK-ED™, and that they perceived it was somewhat helpful in preparing them for work-integrated learning. There were low dropout rates, high attendance at the tutorials, and blind assessment, suggesting the results are credible.

MASK-ED™ simulation may not have been more effective than role play for several reasons. The feedback on performance received by the students was often indirect feedback from the MASK-ED™ character. For example, if the student’s instructions included too much jargon or were not clear then ‘Joyce’ would not complete the task accurately or would ask questions to clarify. The students had the opportunity to self-reflect and debrief with the other educator; however, it was not always possible for this educator to have seen all their practice with the MASK-ED™ character. This may have limited the extent of any feedback. As the students were in groups they didn’t have to interact with the MASK-ED™ character, potentially limiting the amount of deliberate practice and mastery learning undertaken by each student. Curriculum integration and educational and professional context of the classroom-based simulation was identified by some of the students of an area requiring further fine tuning and could have been made more explicit to the students. For example, the Falls tutorial was linked with the lecture material for the neurological condition of Parkinson’s Disease, however ‘Joyce’ did not have Parkinson’s Disease. The MASK-ED™ character was implemented into this tutorial due to the link between aging and falls and the difficulty in training a patient to get off the floor when they have weakness down one side of their body post stroke. Yet some students reported that they did not feel it was appropriate to incorporate MASK-ED™ simulation into this tutorial. Several of these design features warrant further investigation.

Also, the quantity of simulation may not have been enough to elicit a change in performance, relative to usual teaching. MASK-ED™ simulation does not seem to be effective when it is introduced to five out of a potential 32 tutorials. It is possible that more time with MASK-ED™ simulation may be required to influence students’ work integrated learning performance. The inclusion of MASK-ED™ in only five tutorials was related to the resource requirements of MASK-ED™ simulation, i.e., a reliance on trained educators and the time impost for that trained educator, as well as the appropriateness of MASK-ED™ simulation as a strategy for meeting learning objectives. It is recommended that the past medical history and presenting condition of a MASK-ED™ character is not altered once introduced, and therefore it was not appropriate for ‘Joyce’ to have had a stroke in the first neurological unit and then, for example, a spinal cord injury, Parkinson’s Disease, or multiple sclerosis in the second unit of study.

There was a 52% response rate for the satisfaction survey. The students acknowledged that if they were underprepared for the tutorials then they were not ready to practise with the MASK-ED™ character and they may not have benefited from the intervention as intended. Some students felt that the preparation time impacted on their learning, yet others were able to reflect that this is likely more realistic of a real-world situation. Students also reported they felt MASK-ED™ was more beneficial when completed one-on-one, rather in a group. Therefore, it is also possible that MASK-ED™ simulation in a group may not be as effective as when used in a one-on-one encounter. Students reported that it should continue with smaller group sizes. This is also consistent with Bissett et al.’s findings that group-based simulation may take away from the authenticity, especially for less confident students who could opt out of physically practising with the character [24]. It was noted that MASK-ED™ simulation was seen as a potential stressor, however it could be argued that this may be helpful for preparing students for the stress of the clinical environment, particularly when the stressor is moderated appropriately by educators, which can have a positive influence on learning [35]. Based on comments made on the satisfaction survey the benefits of group learning were not adequately explained or demonstrated to the participants.

To date there have been no other randomized trials investigating the effect MASK-ED™ simulation has on work integrated learning of health professional students. Although MASK-ED™ simulation was no better than role play with peers in improving clinical performance, it may have other benefits for preparing students for work integrated learning. Research undertaken by the designers of MASK-ED™ simulation have found that MASK-ED™ simulation has been shown to decrease fear and increase confidence related to nursing skills such as bathing and showering patients [27, 36], and to improve readiness for work integrated learning for nursing students [27]. Medical imaging and sonography students reported finding that MASK-ED™ simulation made learning fun, improved their empathy and communication skills and enabled problem solving and reflection to take place [26]. Students in this study did enjoy the MASK-ED™ simulation, so it may make learning more enjoyable and reduce stress associated with starting work-integrated learning, similar to the results for nursing and medical imaging students, even though there was no effect on actual performance [26, 27].

Post hoc analysis was performed to determine if there were any differences across the groups based on grade point averages (GPAs), or the degree (i.e., postgraduate vs undergraduate). The degree was not an independent variable, and there were no significant differences across all outcome measures. Grade point average did not have an independent effect on work integrated learning results between students in the experimental and control groups when examined using the following GPA levels: low (≤4.5), low–medium (4.51–5.5), medium–high (5.51–6.5), and high (≥6.51) (Tables S1, S2, S3, S4, S5, S6, S7 and S8). There was no significant difference in numbers of students in the experimental and control groups at each GPA level. Noting that the study was powered for a sample size of 120, and there were only 6 students in the low GPA level, there was a trend towards the experimental group performing better in *Communication* during their work integrated learning placement for the students with low GPAs (*p* = 0.09). Further exploration may be warranted to determine whether MASK-ED™ simulation has a role in assisting those students with lower GPAs in preparation for work integrated learning.

## Future research

It is worth considering the use of MASK-ED™ simulation in areas such as remediation for students with poorer performance on practical and written examinations, prior to them commencing work-integrated learning. Commonly students do not receive specific remediation for clinical skills, they will often just repeat their work-integrated learning placement. Exploring the use of targeted practise with a MASK-ED™ character prior to commencing work-integrated learning or following a failed placement may demonstrate that this form of classroom-based simulation results in better clinical performance. This would also have the benefits of alleviating some of the stress that having an underperforming student on placement can have on clinical educators.

## Limitations

This current study was not powered to investigate the difference between the different GPA groups, therefore the trend towards MASK-ED™ simulation being useful for those students with lower GPAs is not a definitive finding. The moderate response rate on the survey was also a limitation of this study. If surveyed, the control group may have provided insights into students’ perceptions of usual teaching. Some of the survey questions may be perceived as biasing, however, this was not the primary outcome of the study, and perceptions of MASK-ED™ have been well documented elsewhere [24, 26, 27]. Students had already been assigned to their tutorial groups and therefore individual students could not be randomized, however based on the characteristics of the students in the experimental and control groups the cluster randomization achieved the result of comparable groups. Another potential limitation in training physiotherapy students in the assessment and treatment of patients with neurological deficits could be the ability of the lecturer playing the role of the MASK-ED™ character to display neurological deficits accurately and consistently, such as upper limb weakness. Differences in educator experience could also have influenced these results.

## Conclusion

MASK-ED™ simulation, when combined with usual teaching, was found to be no more effective than role play with peers on improving physiotherapy students’ clinical performance. Students who participated in classroom-based MASK-ED™ simulation perceived it to be helpful, however most felt they would have liked more time to practise with the MASK-ED™ character. The type, frequency, fidelity, design features, and quantity of classroom-based simulation requires further investigation.

## Supplementary Information

**Additional file 1: Table S1.** Mean (SD) and mean difference (95% CI) and significance for Assessment of Physiotherapy Practice for students with a grade point average ≤ 4.5. **Table S2.** Mean (SD) and mean difference (95% CI) and significance for Assessment of Physiotherapy Practice for students with a grade point average 4.5–5.5. **Table S3.** Mean (SD) and mean difference (95% CI) and significance for Assessment of Physiotherapy Practice for students with a grade point average 5.51–6.5. **Table S4.** Mean (SD) and mean difference (95% CI) and significance for Assessment of Physiotherapy Practice for students with a grade point average ≥ 6.51. **Table S5.** Mean (SD) and mean difference (95% CI) and significance for practical and written examinations for students with a grade point average ≤ 4.5. **Table S6.** Mean (SD) and mean difference (95% CI) and significance for practical and written examinations for students with a grade point average 4.51–5.5. **Table S7.** Mean (SD) and mean difference (95% CI) and significance for practical and written examinations for students with a grade point average 5.51–6.5. **Table S8.** Mean (SD) and mean difference (95% CI) and significance for practical and written examinations for students with a grade point average 5.51–6.5.

## Data Availability

The full data set is available on request to the corresponding author.
