# Supplementary material for: Impact of classroom-based MASK-ED™ (KRS simulation) on physiotherapy student clinical performance: a randomized cluster trial
Source: BMC Med Educ. 2022 Jun 2;22:426. doi: 10.1186/s12909-022-03467-8 (PMC9164409; doi:10.1186/s12909-022-03467-8)
Supplement: Supplementary file 1 — Additional file 1: Table S1. Mean (SD) and mean difference (95% CI) and significance for Assessment of Physiotherapy Practice for students with a grade point average ≤ 4.5. Table S2. Mean (SD) and mean difference (95% CI) and significance for Assessment of Physiotherapy Practice for students with a grade point average 4.5–5.5. Table S3. Mean (SD) and mean difference (95% CI) and significance for Assessment of Physiotherapy Practice for students with a grade point average 5.51–6.5. Table S4. Mean (SD) and mean difference (95% CI) and significance for Assessment of Physiotherapy Practice for students with a grade point average ≥ 6.51. Table S5. Mean (SD) and mean difference (95% CI) and significance for practical and written examinations for students with a grade point average ≤ 4.5. Table S6. Mean (SD) and mean difference (95% CI) and significance for practical and written examinations for students with a grade point average 4.51–5.5. Table S7. Mean (SD) and mean difference (95% CI) and significance for practical and written examinations for students with a grade point average 5.51–6.5. Table S8. Mean (SD) and mean difference (95% CI) and significance for practical and written examinations for students with a grade point average 5.51–6.5. [file 12909_2022_3467_MOESM1_ESM.docx]

Table S1. Mean (SD) and mean difference (95% CI) and significance for Assessment of Physiotherapy Practice for students with a grade point average ≤4.5

| Outcome | GPA ≤4.5 | | | |
| --- | --- | --- | --- | --- |
|  | Exp  (n = 3)  Mean (SD) | Con  (n = 3)  Mean (SD) | Difference between groups  Mean (95% CI) | Significance^a^ |
| APP Communication (verbal and non-verbal) Out of 4 | 2.67 (0.577) | 1.67 (0.577) | -1.00  (-2.309 to 0.309) | 0.099 |
| APP Professionalism  Out of 16 | 11.00 (0) | 8.67 (2.887) | -2.33  (-6.961 to 2.294) | 0.480 |
| APP Assessment  Out of 12 | 7.00 (1.732) | 5.33 (1.155) | -1.67  (-5.004 to 1.670) | 0.197 |
| APP Intervention  Out of 20 | 10.33 (0.577) | 9.33 (2.081) | -1.00  (-4.463 to 2.462) | 0.637 |
| APP Total  Out of 80 | 45.33 (5.132) | 37.67 (9.074) | -7.67  (-24.377 to 9.043) | 0.275 |

^a^Significance determined by Mann-Whitney U test

Exp = experimental group, Con = control group, APP = Assessment of Physiotherapy Practice, SD = Standard deviation, CI = confidence interval

Table S2. Mean (SD) and mean difference (95% CI) and significance for Assessment of Physiotherapy Practice for students with a grade point average 4.5–5.5

| Outcome | GPA 4.51-5.5 | | | |
| --- | --- | --- | --- | --- |
|  | Exp  (n = 20)  Mean (SD) | Con  (n = 20)  Mean (SD) | Difference between groups  Mean (95% CI) | Significance^a^ |
| APP Communication (verbal and non-verbal) Out of 4 | 2.90 (0.718) | 2.60 (0.940) | 0.27  (-0.836 to 0.236) | 0.275 |
| APP Professionalism  Out of 16 | 12.35 (2.907) | 11.60 (2.891) | -0.75  (-2.606 to 1.106) | 0.519 |
| APP Assessment  Out of 12 | 8.15 (1.565) | 7.50 (1.878) | 0.55  (-1.757 to 0.457) | 0.384 |
| APP Intervention  Out of 20 | 13.48 (3.641) | 12.48 (3.997) | 1.21  (-3.45 to 1.448) | 0.662 |
| APP Total  Out of 80 | 56.13 (12.316) | 51.03 (13.914) | 4.16  (-13.512 to 3.3112) | 0.304 |

^a^Significance determined by Mann-Whitney U test

Exp = experimental group, Con = control group, APP = Assessment of Physiotherapy Practice, SD = Standard deviation, CI = confidence interval

Table S3. Mean (SD) and mean difference (95% CI) and significance for Assessment of Physiotherapy Practice for students with a grade point average 5.51–6.5

| Outcome | GPA 5.51-6.5 | | | |
| --- | --- | --- | --- | --- |
|  | Exp  (n = 34)  Mean (SD) | Con  (n = 41)  Mean (SD) | Difference between groups  Mean (95% CI) | Significance^a^ |
| APP Communication (verbal and non-verbal) Out of 4 | 2.85 (0.744) | 3.05 (0.773) | 0.20  (-0.156 to 0.547) | 0.249 |
| APP Professionalism  Out of 16 | 12.15 (2.500) | 12.39 (2.737) | 0.24  (-0.974 to 1.460) | 0.702 |
| APP Assessment  Out of 12 | 7.65 (1.704) | 8.32 (1.809) | 0.67  (-0.145 to 1.485) | 0.115 |
| APP Intervention  Out of 20 | 12.63 (2.762) | 13.46 (3.061) | 0.83  (-0.523 to 2.185) | 0.256 |
| APP Total  Out of 80 | 53.43 (11.140)0. | 55.732 (11.523) | 2.31  (-2.942 to 7.553) | 0.418 |

^a^Significance determined by Mann-Whitney U test

Exp = experimental group, Con = control group, APP = Assessment of Physiotherapy Practice, SD = Standard deviation, CI = confidence interval

Table S4. Mean (SD) and mean difference (95% CI) and significance for Assessment of Physiotherapy Practice for students with a grade point average ≥6.51

| Outcome | GPA ≥ 6.51 | | | |
| --- | --- | --- | --- | --- |
|  | Exp  (n = 5)  Mean (SD) | Con  (n = 6)  Mean (SD) | Difference between groups  Mean (95% CI) | Significance^a^ |
| APP Communication (verbal and non-verbal) Out of 4 | 3.00 (0.707) | 3.33 (0.816) | -0.33  (-1.373 to 0.707) | 0.431 |
| APP Professionalism  Out of 16 | 13.20 (2.168) | 13.83 (2.563) | -0.63  (-3.861 to 2.594) | 0.509 |
| APP Assessment  Out of 12 | 9.20 (1.095) | 8.50 (1.049) | 0.70  (-0.786 to 2.186) | 0.333 |
| APP Intervention  Out of 20 | 15.75 (2.512) | 14.08 (3.073) | 1.67  (-2.143 to 5.476) | 0.359 |
| APP Total  Out of 80 | 63.35 (6.827) | 63.08 (9.489) | 0.27  (-10.917 to 11.451) | 0.647 |

^a^Significance determined by Mann-Whitney U test

Exp = experimental group, Con = control group, APP = Assessment of Physiotherapy Practice, SD = Standard deviation, CI = confidence interval

Table S5. Mean (SD) and mean difference (95% CI) and significance for practical and written examinations for students with a grade point average ≤4.5

| Outcome measure | GPA ≤4.5 | | | |
| --- | --- | --- | --- | --- |
|  | Exp  (n = 3)  Mean (SD) | Con  (n = 3)  Mean (SD) | Difference between groups (95% confidence interval) | Significance |
| Practical Examination 1 (Semester 1) Out of 25 | 13.5  (5.27) | 10.25  (2.38) | -3.25  (-12.52 to 6.02) | 0.385 |
| Practical Examination 2 (Semester 1) Out of 25 | 20.17  (1.26) | 14.91  (3.11) | -5.25  (-10.62 to 0.12) | 0.053 |
| Practical Examination 1 (Semester 2) Out of 15 | 10.58  (0.89) | 7.51  (2.53) | -3.07  (-7.35 to 1.22) | 0.118 |
| Practical Examination 2  (Semester 2) Out of 20 | 15.83  (1.89) | 12.37  (3.13) | -3.47  (-9.34 to 2.40) | 0.176 |
| Written Examination  (Semester 1) Out of 50 | 36.5  (3.97) | 28.11  (1.45) | -8.39  (-15.12 to -1.61) | 0.026* |
| Written Examination  (Semester 2) Out of 40 | 28.87  (1.03) | 21.75  (7.40) | -7.12  (-19.09 to 4.85) | 0.174 |

Exp = experimental group, Con = control group, NI = Neurological Interventions, *p<0.05

Table S6. Mean (SD) and mean difference (95% CI) and significance for practical and written examinations for students with a grade point average 4.51–5.5

| Outcome measure | GPA 4.5.1-5.5 | | | |
| --- | --- | --- | --- | --- |
|  | Exp  (n = 20)  Mean (SD) | Con  (n = 20)  Mean (SD) | Difference between groups (95% confidence interval) | Significance |
| Practical Examination 1 (Semester 1) Out of 25 | 14.51  (4.69) | 15.94  (4.46) | 1.13  (-1.50 to 4.35) | 0.331 |
| Practical Examination 2 (Semester 1) Out of 25 | 19.44  (2.96) | 19.11  (3.19) | -0.33  (-2.30 to 1.65) | 0.740 |
| Practical Examination 1 (Semester 2) Out of 15 | 10.75  (1.81) | 11.19  (2.12) | 0.45  (-0.81 to 1.71) | 0.474 |
| Practical Examination 2  (Semester 2) Out of 20 | 14.57  (1.22) | 14.85  (2.03) | 0.285  (-0.786 to 1.36) | 0.593 |
| Written Examination  (Semester 1) Out of 50 | 30.10  (4.45) | 31.78  (3.93) | 0.79  (-1.90 to 3.47) | 0.558 |
| Written Examination  (Semester 2) Out of 40 | 27.43  (2.77) | 26.03  (4.59) | -1.40  (-3.83 to 1.02) | 0.248 |

Exp = experimental group, Con = control group, NI = Neurological Interventions

Table S7. Mean (SD) and mean difference (95% CI) and significance for practical and written examinations for students with a grade point average 5.51–6.5

| Outcome measure | GPA 5.51-6.5 | | | |
| --- | --- | --- | --- | --- |
|  | Exp  (n = 34)  Mean (SD) | Con  (n = 41)  Mean (SD) | Difference between groups (95% confidence interval | Significance |
| Practical Examination 1 (Semester 1) Out of 25 | 19.54  (3.35) | 17.69  (3.83) | -1.86  (-3.53 to -0.18) | 0.030* |
| Practical Examination 2 (Semester 1) Out of 25 | 20.58  (2.92) | 20.39  (2.62) | -0.19  (-1.47 to 1.08) | 0.765 |
| Practical Examination 1 (Semester 2) Out of 15 | 12.24  (1.95) | 12.30  (1.76) | 0.052  (-0.80 to 0.906) | 0.903 |
| Practical Examination 2  (Semester 2) Out of 20 | 15.88  (1.72) | 15.23  (1.76) | -0.65  (-1.45 to 0.16) | 0.113 |
| Written Examination  (Semester 1) Out of 50 | 32.65  (4.42) | 33.62  (4.94) | 0.67  (-1.51 to 2.85) | 0.541 |
| Written Examination  (Semester 2) Out of 40 | 28.44  (3.71) | 28.52  (3.57) | 0.09  (-1.60 to 1.76) | 0.920 |

Exp = experimental group, Con = control group, NI = Neurological Interventions, *p<0.05

Table S8. Mean (SD) and mean difference (95% CI) and significance for practical and written examinations for students with a grade point average 5.51–6.5

| Outcome measure | GPA 5.51-6.5 | | | |
| --- | --- | --- | --- | --- |
|  | Exp  (n = 5)  Mean (SD) | Con  (n = 6)  Mean (SD) | Difference between groups (95% confidence interval) | Significance |
| Practical Examination 1 (Semester 1) Out of 25 | 21.50  (1.16) | 22.58  (2.22) | 1.08  (-1.42 to 3.59) | 0.353 |
| Practical Examination 2 (Semester 1) Out of 25 | 21.25  (2.82) | 20.79  (2.03) | -0.46  (-3.76 to 2.84) | 0.761 |
| Practical Examination 1 (Semester 2) Out of 15 | 13.92  (0.80) | 13.38  (1.16) | -0.55  (-1.94 to 0.85) | 0.400 |
| Practical Examination 2  (Semester 2) Out of 20 | 16.58  (1.38) | 17.57  (0.62) | 0.99  (-0.70 to 2.67) | 0.147 |
| Written Examination  (Semester 1) Out of 50 | 35.55  (2.09) | 37.66  (3.43) | 2.11  (-1.74 to 5.97) | 0.261 |
| Written Examination  (Semester 2) Out of 40 | 32.74  (3.09) | 33.37  (2.88) | 0.63  (-3.45 to 4.70) | 0.737 |

Exp = experimental group, Con = control group, NI = Neurological Interventions
